# Exploring the potential of civic engagement to strengthen mental health systems in Indonesia (IGNITE): a study protocol

**DOI:** 10.1186/s13033-018-0227-x

**Published:** 2018-08-25

**Authors:** Helen Brooks, Karen James, I Irmansyah, Budi-Anna Keliat, Bagus Utomo, Diana Rose, Erminia Colucci, Karina Lovell

**Affiliations:** 1 Department of Psychological Sciences, Institute of Psychology, Health and Society, University of Liverpool, Liverpool, UK; 2 Centre for Health and Social Care Research, Faculty of Health, Social Care and Education, Kingston and St Georges, London, UK; 3 National Institute of Health Research and Development, Jakarta, Indonesia; 4 Marzoeki Mahdi Hospital, Bogor, Indonesia; 5 Faculty of Nursing, University of Indonesia, Depok, Indonesia; 6 KPSI, Jakarta, Indonesia; 7 Department of Health Services Research, Kings College London, London, UK; 8 Department of Psychology, Middlesex University, London, UK; 9 Division of Nursing, Midwifery and Social Work, School of Health Sciences, Faculty of Biology, Medicine and Health, University of Manchester, Manchester Academic Health Science Centre, Manchester, UK

**Keywords:** Realist methodology, Civic engagement, User involvement, Lived experience, Mental health, Indonesia, Implementation

## Abstract

**Background:**

Indonesia has the highest rate of years of life lost to disability or early death from Schizophrenia than any other country in the world. More than 90% of people with mental illness do not get any treatment and tens of thousands of people with psychosis are illegally detained (‘pasung’) in the family home. Civic engagement, a core part of the recent World Health Organisation global strategy, has the potential to address some of these challenges through the development of person-centered models of care. The aim of the study is to develop a testable systems level, culturally appropriate, civic engagement framework for use in Jakarta and Bogor, Indonesia to strengthen local mental health services.

**Methods:**

A mixed methods study underpinned by a realist approach will be undertaken across four phases in two study sites in Indonesia (Jakarta and Bogor). Phase 1 will explore the use of civic engagement across South East Asia by conducting a systematic review of existing evidence. By surveying 300 mental health professionals, phase 2 will identify the stakeholders, the sources of collaboration and the evidence used by professionals in decision making within local mental health systems and identify potential opportunities for civic engagement within the system. In order to explore the potential use of civic engagement within Indonesian mental health services and identify priorities for a culturally appropriate framework, phase 3 will undertake two focus groups with participants with experience of psychosis or caring for someone with psychosis (n = 20–30). Professionals and other key decision makers in a range of roles across the system at a national (n = 5) and local level (n = 10–15/site) will also take part in semi-structured interviews. Phase 4 will co-produce a civic engagement framework for use in Indonesia by synthesising evidence from phases 1–3 collaboratively with key stakeholders.

**Discussion:**

Civic engagement is a potential way in which health services in low and middle income countries can address the burden of mental health conditions through the development of person-centred models of care. However, such approaches are underexplored in Indonesia. This study will work with local stakeholders to design a testable civic engagement framework for use in mental health services in Indonesia.

**Electronic supplementary material:**

The online version of this article (10.1186/s13033-018-0227-x) contains supplementary material, which is available to authorized users.

## Background

Schizophrenia, the most common psychotic condition, is a major contributor to global morbidity and is ranked 11th among the top 25 causes of disability worldwide [1, 2]. The burden of psychosis is considerable and wide ranging, including reduced life expectancy, social exclusion, poor quality of life, and poor physical and emotional health amongst caregivers [3, 4]. Psychosis has extensive personal and financial consequences for individuals, their families and the economy, estimated at up to 102,396 million dollars nationally per year [5].

In Indonesia, as in most low and middle income counties (LMICs), mental health systems are struggling to meet the needs of people with psychosis and their surrounding communities; more than 90% of people with mental illness do not get any treatment and Indonesia has the highest rate of years of life lost to disability or early death from Schizophrenia worldwide [6]. This, combined with low mental health literacy (poor knowledge of mental health) and high levels of stigma within the general population has resulted in tens of thousands of people being illegally restrained and/or confined (‘pasung’) in the family home [7].

Civic engagement, a core part of the recent WHO global strategy, could help to address these challenges [8]. The World Health Organisation defines civic engagement is ‘a process by which people are enabled to become actively and genuinely involved in defining the issues of concern to them, in making decisions about factors that affect their lives, in formulating and implementing policies, in planning, developing and delivering services and in taking action to active change’. [9].

Derived from social movements such as the Civil Rights Movement, the benefits of civic engagement have been demonstrated across the world, and include improved access to, and quality of care, increased health literacy, reduced stigma, better outcomes for service users and reduced service costs [8, 10]. In a health systems context, civic engagement is a ‘bottom-up’ approach in which service users and their families become actively involved in the design and delivery of health services, also known as ‘co-production’ or ‘user involvement’ [11].

Civic engagement can be enacted at macro, meso and micro levels within a system. For example, through service user membership on national policy committees (macro), user interviewers on clinical recruitment panels (meso) and shared decision-making and joint care planning in routine practice (micro). It recognises lived experience as an important and valuable form of expert knowledge, and so strengthens health systems by using it alongside clinical or scientific expertise in decision making, leading to the development of people-centred services; an ideal for health systems globally [8].

The transformative impact of civic engagement is evident in the Western world where the move from traditional ‘paternalistic’ approaches towards people-centered models of care has largely been attributed to the survivor/user movement [12]. It is particularly important in mental health systems, where the social determinants and impacts of mental health problems, frequently prioritised by service users, are often overlooked by professionals due the dominance of clinical models [13], and where high levels of stigma and coercion mean that the voices of service users and their families often go unheard [14].

Relationships between people’s contributions to society, civic engagement and the functioning of health institutions have become key areas of theoretical and empirical work within the social sciences. The increased salience of civic engagement within the mental health field has been evident over the last five decades due to a combination of factors including deinstitutionalisation, critiques of traditional biomedical models of psychiatry and a move towards consumerism [15]. A burgeoning evidence base demonstrates the benefits of patient and public involvement in service planning and delivery both to health institutions and to the individuals they seek to serve [16]. For example, mental health care plans that have been collaboratively produced between service users and professionals lead to better individual and service level outcomes [17]. Furthermore, involving service users in the design of mental health care has been shown to improve service development, increase service user confidence and enhance staff attitudes towards service users [18].

An examination of the extant theory underpinning civic engagement identifies potential mechanisms through which such activities are thought to impact on individual, service and community level outcomes. At an individual level civic engagement is thought to increase mental health literacy and confidence amongst service users which promotes engagement with services and improves health outcomes [17]. Civic engagement has been shown to improve the health and subsequent quality of life for older people through increased engagement with meaningful activity and increased social interaction [19]. At a systems level, recognising the importance and value of lived experience, strengthens systemic development, improves staff attitudes and increases service user satisfaction through the development of people-centred services [15, 20]. System performance can also be increased through enhanced levels of accountability at all levels of the system instigated by civic engagement [21]. Civic engagement can also reduce stigma and self-stigma within communities by promoting empowerment and social inclusion [22].

Despite the pervasiveness of person-centred care in the academic, political and policy rhetoric [23] and research demonstrating the utility of these approaches in improving the quality of mental health care [20], international literature suggests a sustained failure to effectively implement civic engagement principles within mental health services [18, 24]. Potential reasons for this failure to translate civic engagement into every day mental health practice include no shared definition of civic engagement, the history of coercion and control within services, finite resources, professional resistance, organisational influences and the on-going stigmatisation of mental health service users [25–27]. Such challenges demonstrate the importance of developing an in-depth understanding of the context in which such activities are to be enacted prior to implementation.

As a rapidly developing LMIC, Indonesia’s mental health system is expanding; mental health is becoming a national priority, whilst at a local level clinicians are starting to set up the first community based mental health services and literacy programmes. This early stage of development presents a unique opportunity for civic engagement to shape and strengthen these emerging systems, and ensure that they are designed around the needs and preferences of the people they aim to serve. Yet civic engagement is an under explored area in Indonesia. This protocol was developed collaboratively with local service users, carers and providers and aims to address the priority areas raised by them.

### Theoretical framework

This study will adopt a realist approach, which is a type of theory-driven evaluation set apart by its discrete philosophical underpinnings [28]. It focuses on four linked concepts: mechanism; context; outcome and context–mechanisms–outcome (CMO) configurations, to examine intervention function and contextual variation in intervention effects, i.e. what works, for whom and under what circumstances [29, 30]. In a realist analysis, CMO configurations are developed, tested and validated through an iterative process. A realist approach is particularly useful when examining the transfer of complex social interventions, such as civic engagement, to different settings because of its consideration of context and heterogeneity [28], and has successfully been used in mental health systems previously [31].

This project will use this theoretical framework to develop causal models (CMO configurations) which will explain the feasibility (i.e. contextual barriers and facilitators), mechanisms, and potential impacts (i.e. outcomes) of civic engagement on Indonesian mental health systems, derived initially from a systematic literature review (phase 1) and then validated/further developed during phases 2–4. For example, phase 2, a social network analysis of the stakeholders and evidence implicated in decision making within Indonesian health systems, will give an overview of the local context and identify opportunities for civic engagement. Interviews with key stakeholders (phase 3) will be used to support, refute or modify CMO configurations identified during phase 1 and develop a culturally appropriate framework for civic engagement in Indonesia. This will then be presented to participants at a series of synthesis workshops where the framework will be further refined (phase 4).

### Patient and public involvement

This project was developed in collaboration with our project partner, Komunitas Peduli Skizofrenia Indonesia (KPSI). KPSI is a user-led charity, set up and run by people with a diagnosis of schizophrenia and the family members who care for them.

KPSI and our contacts in community health services at our study sites have identified a group of service users and carers who will act as an advisory group for this project. They will review key documents (e.g. questionnaire and interview schedules) throughout the study and will meet at least three times during the course of the project. A research methods course for service users and carers, was provided to researchers and members of the advisory group in January 2018, and will be supplemented with informal mentorship from the research team. Two members of the advisory group will receive bespoke training in qualitative methods to allow them to contribute to the data analysis in phase 3 of the study.

## Methods

This mixed-methods study involves four phases. Data collection will take place across two study sites in Indonesia; Jakarta and Bogor. These sites were selected because of pre-identified collaborators in each area and due to their differing geographical, economic and urban–rural contexts, and differences in the standard and development of local mental health systems. All data will be collected by Indonesian co-applicants and researchers and analysed collaboratively within the wider research team. The protocol has been prepared using the Standard Protocol Items: Recommendations for Interventional Trials guidelines (SPIRIT, see Additional file 1).

### Primary aim

To develop a testable systems level, culturally appropriate, civic engagement framework for use in Jakarta and Bogor, Indonesia to strengthen local mental health services.

### Research objectives

1. To understand current civic engagement approaches across South-East Asia (phase 1);
2. To examine exemplars of good practice in relation to the use of civic engagement in South-East Asia (phase 1);
3. To understand how decisions about healthcare design and delivery are currently made within Jakarta and Bogor and identify the key stakeholders involved, and information used, in this process (phase 2);
4. To identify key opportunities for civic engagement within the mental health care system (phase 2);
5. To examine stakeholders’ perspectives (e.g. users, carers, clinicians, policy makers, NGOs) on civic engagement (phase 3);
6. To examine the micro, meso and macro level facilitators and barriers to the development of people centred mental health care systems through civic engagement in Indonesia (phase 3);
7. To develop the optimal strategy for future testing of a civic engagement programme in mental health care systems in Indonesia phase 4).

### Phase 1: Systematic review (months 1–6)

#### Aim

To identify current civic engagement approaches in mental health services previously implemented in South-East Asia (including exemplars of good practice) and critically appraise and synthesize current evidence around the context, mechanisms and outcomes of these approaches. These data will be used to refine our approach to phase 3 and will be integrated with the findings from phases 2 and 3, using the realist approach, to develop a testable framework for civic engagement in Jakarta and Bogor. The review will answer the following questions:

1. What types of civic engagement approaches in mental health services have been previously implemented in South-East Asia?
2. What is currently known about the context, mechanisms and outcomes of such approaches (identify exemplars of good practice)?
3. What is the extent and quality of the evidence demonstrating the effectiveness of civic engagement approaches in mental health services in South-East Asia?

#### Method

A realist review of published quantitative and qualitative research studies and unpublished grey literature (it is envisaged that relevant work undertaken by NGOs may be unpublished). The methods of this systematic review are informed by PRISMA (Preferred Reporting Items for Systematic Reviews and Meta-Analyses) guidelines [32] and RAMESES quality standards for realist synthesis [33].

Studies will not be excluded on quality, date of publication or sample size. Authors will be contacted for full version of articles where they are only accessible in abstract form.

#### Developing a realist program theory

Following RAMESES guidance [33], through an initial scoping review of the literature, and in consultation with the research team, we have developed an initial realist program theory which has identified the key components of civic engagement, and the ways in which it is expected to work (see “Background”). This theory will be further refined during the systematic review to develop a realist program theory to be used in the remainder of the study.

#### Search strategy and data sources

##### Published studies

For peer-reviewed publications the search strategy will be informed by published reviews, the initial programme theory and theoretical underpinnings, discussion with the wider project team and consideration of MeSH terms. Search terms will be piloted in three online databases. Papers identified through piloting will be assessed for additional terms, subject headings and key words with the aim of further refining the search strategy. Agreed search terms will be combined using the Boolean operator ‘OR’ and across components using ‘AND’. Electronic database searches will be undertaken from the earliest record using Ovid, Global Health Archive, CINAHL, Embase, Health Management Information Consortium, Medline, Medline, Scopus, PsychInfo, Social Science Full Text, and Web of Science.

The reference lists of included papers will also be manually searched for relevant papers. A Google Scholar alert will be created to identify research published during the course of the review.

##### Unpublished grey literature

A grey literature search plan will be developed and implemented by Indonesian co-applicants to incorporate the four different search strategies outlined below. The search plan will be developed in partnership with the wider project team and will outline the resources, search terms, websites, and limits to be used prior to conducting the search [34].

1. Grey literature databases, including institutional repositories, relevant organisations, the New York Academy of Medicine Grey Literature Report, and OpenGrey.
2. Customized Google searches, for documents published online.
3. Targeted websites: a list of relevant organisations will be compiled, in partnership with the wider research team and in consultation with experts in the field (see below). Each website will be searched for potentially relevant documents.
4. Consultation with experts: a range of experts in this field will be contacted via email, including expert librarians, academics (from Higher Education Institutes within Indonesia, the Research and Development Centre and the Ministry of Health), third sector representatives, and those involved in implementing civic engagement or co-production initiatives who will be asked to identify any potentially relevant documents or information sources.

##### Eligibility assessment

Once complete, results from the online searches will be uploaded to Endnote before removing duplicates and exporting the data management software Covidence [35]. The first stage of the review process will involve double screening for eligibility (see below for a list of inclusion and exclusion criteria) at the level of title and abstract for published studies, and abstracts, executive summaries or table of contents for grey literature (since abstracts often do not feature in grey literature documents). An independent reviewer will review the excluded references for validity purposes. Inclusion/exclusion conflicts will be resolved by a third reviewer. Full texts will be screened for inclusion by two independent reviewers and conflicts will be resolved by consensus. Acceptable concordance will be predefined at 90% [36].

#### Eligibility criteria

Inclusion criteria include studies undertaken in South-East Asia (Brunei, Burma, Cambodia, Timor-Leste, Indonesia, Laos, Malaysia, Philippines, Singapore, Thailand, Vietnam, Christmas Island, Cocos Island) related to the use of civic engagement approaches in mental health services. Exclusion criteria include those not accessible online or via inter-library loan and those published only in abstract form.

#### Data extraction

A framework will be created in Microsoft Excel for the purpose of data extraction, which will include categories for context, mechanisms and outcome [37]. Where data are available from quantitative measures, these will be extracted. Qualitative themes related to the study questions will be extracted along with relevant direct quotations. Where data are not available in the document, authors will be contacted by email to request relevant data.

#### Data synthesis

If data allows, meta-analyses will be conducted using random-effects modelling to provide measures of pooled effects and a meta-regression of intervention moderators will be undertaken to examine relationships between intervention components and outcomes.

Quantitative and qualitative data will be synthesised and used to amend the initial program theory, through an iterative, narrative approach, appropriate for realist reviews combining both qualitative and quantitative evidence [38]. The final outcome will be a new CMO configuration which will describe how, in what ways, and in which contexts civic engagement impacts mental health systems in South-East Asia.

### Phase 2: Social network analysis (months 3–6)

#### Aim

To identify the main stakeholders, sources of collaboration, and evidence used by people in decision making within local mental health systems, and opportunities for civic engagement within the system.

#### Methods

An online survey and systems level social network analysis will give in-depth insight into the structure and operation of local health systems. Interpersonal relations will be captured and analysed (quantitatively). This analysis will provide a visual representation of the networks and social structures underlying interactions between clinicians, service users, carers, managers and policy makers to help identify opportunities for civic engagement, and identify key people who could act as facilitators, at various levels within the system [39]. This will help us to understand how civic engagement could be implemented within current mental health service provision.

#### Participants and sampling

Participants will include mental health professionals, managers, policy makers and members of third sector organisations involved in mental health decision-making at macro, meso or micro levels across the mental health system. This will include (i) national-level decision making (macro), e.g. around mental health policy, funding and commissioning and quality assurance (ii) local service-level decision making at each study site (meso), e.g. around service design and delivery, governance and evaluation, staff recruitment and training and (iii) individual-level care planning decisions (micro) within these services.

#### Recruitment

Working with Indonesian co-applicants and local service managers a database of people likely to be involved in mental health decision-making will be compiled, including contact details (email addresses and telephone numbers) for each individual. Local collaborators have estimated this number to be approximately 300. Participants will be provided with a link to the survey with the consent form and information sheet via email or Whatsapp message, and will be invited to contact the researcher if they have any questions.

Data collection will be open for a minimum of 2 weeks, allowing potential participants plenty of time to make a decision about whether or not to take part. Non-responders will be followed up by telephone. To ensure all relevant decision makers are included a referral sampling technique will be used whereby participants will be asked to nominate other individuals and organisations involved in mental health decision making [40].

#### Data collection

Data will be collected through an online survey (SelectSurvey), which will ask participants to name individuals involved in decision making and the sources of information they use when making decisions. It will include questions about involvement of people with psychosis and their families. The survey will also collect the following data: a rating of importance/influence of the individual and/or information used, characteristics of participant and named individual (e.g. role, institution), and type of interaction (e.g. committee meeting, consultation). Where relevant, participants will be asked to choose from a list of possible responses (including a free text box) [41].

#### Data analysis

Data will be analysed using social network analysis. This analysis is concerned with explaining social phenomena (e.g. decision making) using the structural and relational features of the network of individuals involved. For example, this approach can be used to identify what information is used in decision-making, how it is exchanged and who is included or excluded from the process. Data software such as UCINet [42] will be used to map links between named individuals and the centrality of participants (frequency of nomination). This analysis will be supplemented by a documentary analysis of existing policy and practice guidelines and care planning tools relating to involvement of service users and carers in the design and delivery of care.

Analysis will explore:

- The main types of people and sources of information implicated in decisions (frequency analysis).
- The perceived level of influence of individuals, information and interaction types.
- Connectedness, including centrality.
- Direction of flow (i.e. information exchange).

### Phase 3: Stakeholder interviews (months 7–14)

#### Aim

To explore views of civic engagement amongst key stakeholders across the mental health system and how it might work for people in Indonesia, and to test and further develop CMO configurations identified during phase 1.

#### Methods

##### Participants and sampling

Professionals and other key decision makers in a range of roles across the system at a national (n = 5) and local level (n = 10–15/site) will be sampled from the map produced in phase 2 (maximum variation sampling will guide sample size [43]). At national level this will include government ministers, policy makers, and leaders of third sector organisations, and at a local level, service directors, senior management, frontline clinical staff and community leaders (including community volunteers such as cadres and professionals within primary and secondary care). Interviews or focus groups (depending on preferences) will also be conducted with 10–15 service users and carers at each of the study sites. This will allow us to explore different elements of the programme theory and develop our understanding of the mechanisms through which civic engagement might lead to different outcomes within Jakarta and Bogor.

##### Recruitment

Service users and carers will be recruited through KPSI and community health services at each study site. Adverts detailing the time and date of focus groups will be displayed in community venues. The advert will include contact details for a local researcher should potential participants wish to obtain more information on the study or request a one-to-one interview.

Professionals and other key stakeholder will receive an email/Whatsapp invitation to take part in the study with a study information sheet. Those who are interested in taking part in the study will contact the study team directly to arrange a one-to-one interview. Posters advertising the study will also be displayed in local hospital sites in both Bogor and Jakarta.

Researchers will be given in-depth training on the process of obtaining informed consent and receive on going supervision over the course of the study. Informed written consent will be obtained from participants prior to interviews and focus groups taking place and continually assessed during study activities.

#### Data collection

Data will be collected through semi-structured in-depth interviews and focus groups. The interview/focus group schedules will be developed from our initial programme theory and drawing on data from phases 1 and 2. Schedules will aim to give further insight into findings about current interactions between services, and service users and their families (from phase 2), challenges within the mental health system, stakeholder views of civic engagement and what this means to them, the potential macro, meso and micro level mechanisms and impacts of this approach and the possible barriers and facilitators to implementation.

Interviews and focus groups will be conducted by researchers and research students from the University of Indonesia and service users and carers all of whom will be provided with training and supervision. Interviews will be undertaken in a private room and focus groups will be undertaken in accessible community venues. Focus groups will include one facilitator, one note taker and an additional researcher to support any participants who may need support during the group.

Interviews and focus groups will be digitally audio-recorded using an encrypted Dictaphone and transcribed.

#### Data analysis

Data from interviews and focus groups will be analysed using thematic analysis, following the six stage process outlined by Braun and Clarke [44], using the analysis software NVivo [45]. We will use a flexible approach incorporating inductive and deductive approaches [46]. The deductive thematic framework will include higher-level a priori themes drawing on our program theory and theoretical underpinnings, including codes for context, mechanisms and outcomes. Our programme theory will therefore be used as a tool to identify and contrast emerging themes during the analytical process in line with realist evaluation guidelines. Our analysis will also elicit detailed descriptions of views of civic engagement and barriers and facilitators to implementation within Indonesia’s mental health system, from a range of different perspectives, which will be used to further refine our CMO models, and generate theory around the acceptability of civic engagement within these contexts.

#### Trustworthiness of data

Various strategies will be employed to ensure trustworthiness of data:

- Detailed field notes will be kept for each group/focus group.
- Preliminary findings will be presented to the PPI advisory group to ensure interpretations remain grounded in the data.
- Translations will be validated by a bilingual individual unrelated to the study and a proportion will be back-translated to ensure correct interpretations [47].
- An audit trail will be kept of various iterations of the thematic framework to ensure transparency of the analytical process.
- Service users and carers will be involved in the process of analysis.

### Phase 4: Synthesis workshops (month 16)

#### Aim

To:

i. Co-produce a testable, culturally appropriate civic engagement framework and implementation strategy for use in Jakarta and Bogor.
ii. Capture stakeholder feedback on our study findings (including via creative visual methods) and identify key questions/topic areas for a larger evaluation of this approach.
iii. Increase stakeholder knowledge of research and civic engagement.
iv. Foster communication and collaboration between professionals and service users and their families.

#### Methods

Service users, carers and professionals along with other key local stakeholders (e.g. service managers and third sector organisations) will be invited to attend a synthesis workshop. The workshop will be held in a community venue. All attendees will be given written information about what the workshop will involve.

The event will comprise a presentation and discussion of the programme theory, research findings, and proposed CMO configurations followed by mixed group activities, using creative methods, to develop a local civic engagement framework and implementation strategy. The framework will take the form of a logic model, outlining the inputs, activities, outputs and impacts of the programme. In this final iterative stage, we will further refine CMO configurations in consultation with local experts and select the most robust and plausible explanations of how civic engagement activities can be used to improve health services in the Indonesian context. These final CMO configurations will then be compared to the initial programme theory which will be modified where necessary.

Visual methods (observational film and video-interviews), will be used to capture important and impactful thoughts and messages amongst stakeholders that will support civic engagement education and awareness activities in Indonesia [48].

#### Outputs/dissemination

Our findings will be disseminated to academic audiences through publications in peer reviewed journals. Briefing papers outlining key research findings will be co-produced with advisory group members which will be accessible to non-academic audiences, and sent directly to all participants. The framework and educational video will be made freely available online.

Our findings will be presented at national and international conferences. Service user and carer co-applicants will be invited to co-present study findings.

Study specific Facebook and Twitter accounts will be set up and social media campaigns will be used to publicise our research and engage with key stakeholders outside of academia. These platforms will be used to disseminate key research outputs, such as our film, open access publications and briefing papers, study reports and co-produced civic engagement strategies.

## Discussion

An on going challenge facing mental health services in LMICs is to reduce the burden associated with the experience of mental health conditions. Indonesia has the highest rate of years of life lost to disability or early death from Schizophrenia than any other country in the world. Civic engagement has the potential to address this challenge through the development of person-centred models of care. However, this is an underexplored and under researched area within Indonesia.

This study will use mixed methods, underpinned by a realist approach, to explore the potential of civic engagement to strengthen mental health systems in Indonesia. The results will be used to develop a testable, systems level culturally appropriate civic engagement framework to be used as a mechanism to promote service user and carer involvement in the design and delivery of mental health care and strengthen emerging health systems.

### Strengths and limitations

This study gains its strengths from the partnership between UK and Indonesian researchers, from the in-depth nature of its design which incorporates both qualitative and quantitative components and the utilisation of a realist approach to examine the transfer of civic engagement principles to the South-Asian context. Patient and public involvement is also central to the research design and proposed undertaking of the study.

This exploratory study will only recruit participants from two geographical locations within Java (Jakarta and Bogor). It therefore may not be possible to fully transfer findings to participants in other areas of Indonesia.

The application of critical realism in research can be a challenge as there are no strict methodological rules to follow, and a lack of detailed guidance around what a realist approach to data processing and analysis should look like in practice [46, 49]. Furthermore, development of theory (CMO configurations) can be difficult if there is a lack of relevant research in the area of interest, or if evaluations or descriptions of existing approaches do not explicitly discuss the underlying theory [49].

## Additional files

**Additional file 1.** SPIRIT 2013 Checklist: Recommended items to address in a clinical trial protocol and related documents.

**Additional file 2.** Ethical approval from the University of Liverpool.

**Additional file 3.** Ethical approval from the University of Indonesia.

**Additional file 4.** Award letter from the Medical Research Council.

## Abbreviations

CMO: context, mechanism, outcome
EQUIP: enhancing the quality of user/carer involvement in mental health care planning
KPSI: Komunitas Peduli Skizofrenia Indonesia
LMIC: low middle income country
NGO: non-government organisation
NICE: National Institute for Clinical Excellence
NIHR: National Institute for Health Research
PPI: patient and public involvement
PRISMA: Preferred Reporting Items for Systematic Reviews and Meta-Analyses
RAMESES: realist and meta-narrative evidence syntheses—evolving standards
RATS: Qualitative Research Review Guidelines
WHO: World Health Organisation
