# Supplementary figures and images for: Exploring the potential of civic engagement to strengthen mental health systems in Indonesia (IGNITE): a study protocol
Source: Int J Ment Health Syst. 2018 Aug 25;12:49. doi: 10.1186/s13033-018-0227-x (PMC6109339; doi:10.1186/s13033-018-0227-x)

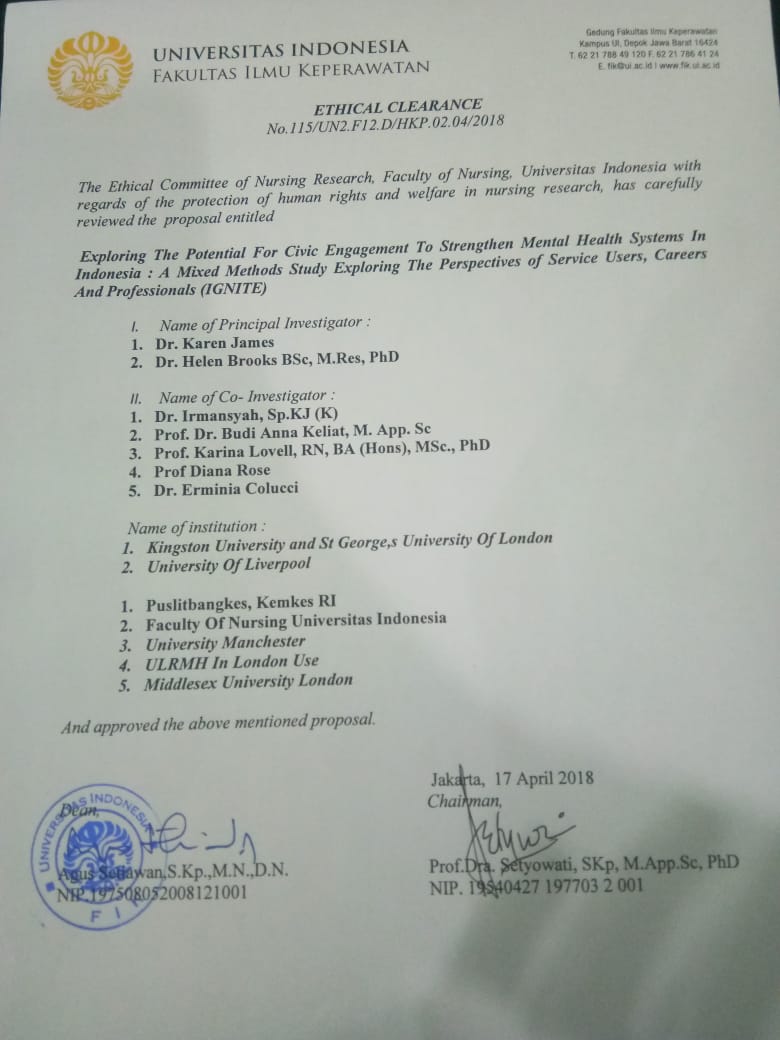

Supplement: Supplementary file 3 — Additional file 3. Ethical approval from the University of Indonesia. [file 13033_2018_227_MOESM3_ESM.jpeg]
