# Supplementary material for: Exploring the potential of civic engagement to strengthen mental health systems in Indonesia (IGNITE): a study protocol
Source: Int J Ment Health Syst. 2018 Aug 25;12:49. doi: 10.1186/s13033-018-0227-x (PMC6109339; doi:10.1186/s13033-018-0227-x)
Supplement: Supplementary file 4 — Additional file 4. Award letter from the Medical Research Council. [file 13033_2018_227_MOESM4_ESM.pdf]

Head of Department
Research Finance
University of Liverpool
2nd Floor Block D, Waterhouse Buildings
Liverpool United Kingdom
L69 3GL

Grant Ref: MR/R003386/1

Date: 8 December 2017

Dear Head of Department

GRANT OFFER: Research Grant, Joint Funded Initiatives Full
GRANT TITLE: Exploring the potential of civic engagement to strengthen mental health systems in Indonesia.

The MRC is offering a grant towards the cost of the above project, subject to the terms and conditions set out below.

Return of the 'Offer Acceptance' will be taken as acceptance of the grant on the terms stated. If you are unable to accept the grant you should return a 'Decline' confirmation as soon as possible. Upon receipt of the 'Offer Acceptance' a 'Start Confirmation' request will be issued.

Grants are cash limited and expenditure against the grant must not exceed the value awarded apart for reasons stated in the standard terms and conditions.

Please note copies of this letter have not been sent to the grant holder and co-investigators (as appropriate); it is your responsibility to distribute copies as is necessary.

Yours faithfully

Grants Pre Award Team
RCUK Grants
A service provided on behalf of MRC

Organisation: University of Liverpool

Grant Holder: Dr Helen Brooks

Grant Title: Exploring the potential of civic engagement to strengthen mental health systems in Indonesia.
Starts: 1 August 2017 **Ends:** 31 January 2019 **Duration:** 18

GRANT VALUE

Funds Awarded

	Authorised FEC (£)			RC Contribution (£)			
	net	Indexation	Total	net	Indexation	Total	% FEC
DI - T&S	16,500	179	16,679	12,210	132	12,342	74
DI - Other Costs	8,700	94	8,794	6,438	70	6,508	74
DA - Investigators	25,962	281	26,243	19,212	208	19,420	74
DA - Estate Costs	3,194	35	3,229	2,364	26	2,389	74
Indirect - Indirect Costs	16,953	183	17,136	12,545	136	12,681	74
Exception - Other Costs	51,369	556	51,925	51,369	556	51,925	100
**Total Value of Award**	**122,678**	**1,328**	**124,005**	**104,137**	**1,127**	**105,264**	

Cost of Access to Facilities 0
(Funds not awarded to Grant Holding Organisation)

STAFF

Staff Summary

	Authorised FEC net	RC Contribution net	Number Of Staff Months
Investigator	25,962	19,212	5

Staff and DI Investigator Details

Start Date	End Date	Duration	FTE Percent	Name or Post Identifier	Summary Fund Heading	Authorised Cost (Excluding Indexation)

DA Investigator Details

Average Hours/week	Name or Post Identifier
4.5	Dr H L Brooks
0	Dr E Colucci
0	Dr I Irmansyah
3	Dr K K James
0	Professor B Keliat
.2	Professor K Lovell
1.9	Professor D Rose

EQUIPMENT DETAILS

Description	Delivery Date	Country Of Origin	Total Value

FACILITY AND SERVICE DETAILS

Facility	Cost of Access	Number of Units

PROJECT PARTNERS

Organisation	Department	Last Name	First Name	In Kind Value(£)	Monetary Value (£)
KPSI (Community Care Schizophrenia Ind)	Head office	Utomo	Bagus	0	0

GRANT ADDITIONAL INFORMATION

GRANT CONDITIONS

CALL CONDITIONS

RESEARCH COUNCIL CONDITIONS

SCHEME CONDITIONS

Terms and Conditions of Research Council fEC Grants
Contents

Introduction
Terms and Conditions of Research Council fEC Grants
Definitions
Data Protection Regulations
Freedom of Information Act and Environmental Information Regulations

Grant Conditions
RGC 1 Accountability & Responsibilities of the Research Organisation
RGC 2 Research Governance
RGC 2.1 Research Ethics
RGC 2.2 Use of Animals in Research
RGC 2.3 Medical Health Research
RGC 2.4 Health and Safety
RGC 2.5 Misconduct and Conflicts of Interest
RGC 3 Use of Funds
RGC 4 Starting Procedures
RGC 5 Changes in Research Project
RGC 6 Transfers of Funds between Fund Headings
RGC 7 Extensions
RGC 8 Staff
RGC 9 Maternity, Paternity, Adoption and Parental Leave
RGC 10 Sick Leave
RGC 11 Equipment
RGC 11.1 Procurement of Equipment
RGC 11.2 Ownership of Equipment
RGC 11.3 Use of Equipment
RGC 11.4 Use of Equipment Funds
RGC 11.5 Equipment.Data
RGC 12 Transfer of a Grant to another Research Organisation
RGC 13 Change of Grant Holder
RGC 14 Annual Statement

RGC 15 Expenditure Statements
RGC 16 Disclosure and Inspection
RGC 17 Reporting on the conduct and results of research
RGC 18 Sanctions
RGC 18.1 Contact Sanctions
RGC 18.2 Organisation Sanctions
RGC 19 Public Engagement
RGC 20 Exploitation and Impact
RGC 21 Research Monitoring and Evaluation
RGC 22 Publication and Acknowledgement of Support
RGC 23 Disclaimer
RGC 24 Status

Introduction

Terms and Conditions of Research Council fEC Grants

These terms and conditions relate to grants, comprising Research Grants and Fellowships, costed and funded on the basis of full economic costs (fEC), calculated in accordance with the TRAC methodology (universities and other higher education bodies) or by an equivalent methodology by other Research Organisations.Grants awarded by the Research Councils are made to Research Organisations on the basis of this single set of core terms and conditions. The Research Councils are:

-Arts and Humanities Research Council (AHRC)
-Biotechnology and Biological Sciences Research Council (BBSRC)
-Economic and Social Research Council (ESRC)
-Engineering and Physical Sciences Research Council (EPSRC)
-Medical Research Council (MRC)
-Natural Environment Research Council (NERC)
-Science and Technology Facilities Council (STFC)

Individual Councils may add additional conditions to the grant to reflect the particular circumstances and requirements of their organisation, or the nature of a particular grant. Acceptance of a grant constitutes acceptance of both the core conditions and any additional conditions. These conditions also apply to, activities subcontracted to 3rd parties. These conditions cannot be waived or varied without the consent of the awarding Research Council. Any request by the grant holder to the council to vary these terms and conditions must be submitted through the Je-S grants maintenance facility and approved in writing by someone authorised to do so on behalf of the Council.

The Research Councils reserve the right to vary these terms and conditions the latest version is available on the RCUK website here: http://www.rcuk.ac.uk/funding/grantstcs

Definitions

Research Council: any of the bodies listed above.

Grant: support for a proportion of the full economic costs of a project. A Grant may be either a Research Grant or a Fellowship;

Research Grant: a contribution to the costs of a stated research project which has been assessed as suitable for funding through the procedures established by the relevant Research Council.

Fellowship Grant: an award made through a fellowship competition providing a contribution to the support of a named individual. It covers the cost of the time dedicated by the fellow to their personal research programme, and may or may not include research support costs.

Grant Holder: the person to whom the grant is assigned and who has responsibility for the intellectual leadership of the project and for the overall management of the research. The Grant Holder is either the Principal Investigator (in the case of a Research Grant) or a Research Fellow (in the case of a Fellowship Grant).

Co-Investigator: a person who assists the Grant Holder in the management and leadership of a project.

Research Organisation: the organisation to which the grant is awarded and which takes responsibility for the management of the research project and the accountability of funds provided.

Third Party: any person/organisation to which the award holding RO passes on any of the grant funds awarded by the RC.

Full Economic Costs (fEC): a cost which, if recovered across an organisation's full programme, would recover the total cost (direct, indirect and total overhead) including an adequate recurring investment in the organisation's infrastructure.

Directly Incurred Costs: costs that are explicitly identifiable as arising from the conduct of a project, are charged as the cash value actually spent and are supported by an audit record.

Directly Allocated Costs: the costs of resources used by a project that are shared by other activities. They are charged to projects

on the basis of estimates rather than actual costs and do not represent actual costs on a project-by-project basis.

Indirect Costs: non-specific costs charged across all projects based on estimates that are not otherwise included as Directly Allocated Costs. They include the costs of the Research Organisation's administration such as personnel, finance, library and some departmental services.

Exceptions: Directly Incurred Costs that Research Councils fund at 100% of fEC. subject to actual expenditure incurred, or items that are outside fEC.

Transparent Approach to Costing (TRAC): an agreed methodology used by universities and other higher education bodies for calculating full economic costs.

Funding Assurance Programme: a programme of visits and office-based tests to seek assurance that grant funds are used for the purpose for which they are given and that grants are managed in accordance with the terms and conditions under which they are awarded.

Data Protection Regulations

The Research Councils will use information provided on the grant proposal for processing the proposal, the award of any consequential grant, and for the payment, maintenance and review of the grant. This may include:

-Registration of proposals. Operation of grants processing and management information systems.
-Preparation of material for use by referees and peer review panels.
-Administration, investigation and review of grant proposals.
-Sharing proposal information on a strictly confidential basis with other funding organisations to seek contributions to the funding of proposals.
-Statistical analysis in relation to the evaluation of research and the study of trends.
-Policy and strategy studies.

To meet the Research Councils' obligations for public accountability and the dissemination of information, contents of funded research proposals will also be made available on the Research Councils' websites and other publicly available databases, including Gateway to Research, and in reports, documents and mailing lists.

After completion of the grant, the Research Council may contact the Grant Holder concerning funding opportunities or events, or for the purposes of evaluation. In some instances, the Research Council may wish to authorise an affiliate organisation to contact the Grant Holder on its behalf. It is assumed that, by agreeing to these terms and conditions, the Research Organisation consents to this on behalf of the Grant Holder, but if the Grant Holder prefers not to be contacted in this way, he or she should state this to the Research Council. Grant Holders may choose to opt out at any point, provided they comply with all other terms and conditions associated with the grant.

Freedom of Information Act and Environmental Information Regulations

Attention is drawn to the provisions of the Freedom of Information Act 2000 (FOIA) and the Environmental Information Regulations (EIRs). Research Councils have issued Publication Schemes which set out the types of information publicly available on their websites or published as documents. In addition, Research Councils have an obligation to respond to specific requests and may be required to disclose information about or provided by Research Organisations. In some cases the Research Council may consult the Research Organisation before disclosure, but it is under no obligation to do so. If a Research Organisation considers that any information it provides to a Research Council would be subject to an exemption under FOIA or the EIRs it should clearly mark the information as such and provide an explanation of why it considers the exemption applies and for how long. The Research Council will consider this explanation before disclosure, but it is not obliged to accept it as binding.

Where a Research Council determines that a Research Organisation is holding information on its behalf that it requires in order to comply with its obligations under FOIA or EIRs, the Research Organisation undertakes to provide access to such information as soon as reasonably practicable on request of the Research Council and in any event within 5 working days.

In some cases Research Organisations may be directly responsible for complying with FOIA and the EIRs; in such cases the Research Councils accept no responsibility for any failure to comply by the Research Organisations.

Grant Conditions

RGC 1 Accountability & Responsibilities of the Research Organisation

- The Research Organisation is accountable for the conduct of the research, the use of public funds and for ensuring the proper financial management of grants. These obligations apply wherever the research is carried out; either at the Research Organisation or a collaborating organisation or other third party.

-The Research Organisation must ensure that funds are spent in a way that is consistent with the purpose and conditions of the award.

-The Research Organisation is responsible for the timely and accurate submission of all expenditure statements and reports required by the award.

-The Research Organisation shall ensure that it carries out appropriate due diligence on any third parties used to deliver any part of the work funded by the grant and shall ensure in particular, that activities carried out by such third parties comply with these terms and conditions. The Research Organisation shall provide the Research Council on request with details of expenditure of the Grant by any third party .

- The Research Organisation must ensure that any part of the Full Economic Cost of the project not funded by the Research Council grant is committed to the project before it starts.

- The Research Organisation must ensure that the Grant Holder and co-investigators are made aware of their responsibilities and that they observe the terms and conditions of grants.

- The Research Organisation must ensure that the research supported by the grant complies with all relevant legislation and Government regulation, including that introduced while work is in progress. This requirement includes approval or licence from any regulatory body that may be required before the research can commence.

- The Research Organisation is expected to adopt the principles, standards and good practice for the management of research staff set out in the 2008 Concordat to Support the Career Development of Researchers, and subsequent amendments.

The Research Organisation must create an environment in which research staff are selected and treated on the basis of their merits, abilities and potential. It must ensure that reliable systems and processes are in place so that the principles of the Concordat are embedded into practice within the Research Organisation. It must ensure compliance with all relevant legislation and Government regulation, including any subsequent amendments introduced while work is in progress.

- The Research Organisation is responsible for compliance with the terms of the Equality Act 2010 including any subsequent amendments introduced while work is in progress; and for ensuring that the expectations set out in the RCUK statement of expectations for equality and diversity are met.

-The Research Organisation is expected to adopt the principles, standards and good practice for public engagement with research set out in the 2010 Concordat for Engaging the Public with Research: http://www.rcuk.ac.uk/pe/Concordat/

-The Research Organisation shall comply with European Union state aid law in their own uses of Research Council funding. In the case of any breach of state aid law the Research Council may be required to recover all or some funding, together with interest. The Research Council may also be required to withhold funding or aspects of funding where the Research Organisation is subject to a state aid inquiry or which has an outstanding recovery notice against it.

- The Research Organisation must create an environment in which public engagement is valued, recognised and supported. It must ensure that reliable systems and processes are in place so that the principles of the Concordat are embedded into practice within the Research Organisation.

- The Research Organisation must appoint a Research Fellow as an employee for the full duration of the award.

- The Research Organisation must integrate the Research Fellow within the research activities of the host department, whilst ensuring that he or she is able to maintain independence and focus on their personal research programme.

-The Research Organisation must notify the Research Council of any change in its status, or that of the Grant Holder, that might affect the eligibility to hold a grant.

- The Research Organisation must ensure that the requirements of the Employing Organisation under the Department of Health's Research Governance Framework for Health and Social Care (or equivalent) are met for research involving NHS patients, their organs, tissues or data, and that the necessary arrangements are in place with partner organisations. Where it also accepts the responsibilities of a Sponsor(as defined in the Governance Framework), it must also ensure that the requirements for Sponsors are met.

- The Research Organisation must ensure that adequate business continuity plans are in place to ensure that operational interruptions to the research are minimised.

-The Research Organisation must retain all accounting information relating to the Grant for the current financial year plus the subsequent six years after the submission date of the final expenditure statement.

RGC 2 Research Governance

It is the responsibility of the Research Organisation to ensure that the research is organised and undertaken within a framework of best practice that recognises the various factors that may influence or impact on a research project. Particular requirements are to ensure that all necessary permissions are obtained before the research begins, and that there is clarity of role and responsibility among the research team and with any collaborators. The Research Councils expect research to be conducted in accordance with the highest standards of research integrity and research methodology.

RGC 2.1 Research Ethics

The Research Organisation is responsible for ensuring that ethical issues relating to the research project are identified and brought

to the attention of the relevant approval or regulatory body. Approval to undertake the research must be granted before any work requiring approval begins. Ethical issues should be interpreted broadly and may encompass, among other things, relevant codes of practice, the involvement of human participants, tissue or data in research, the use of animals, research that may result in damage to the environment and the use of sensitive economic, social or personal data.

RGC 2.2 Use of Animals in Research

Wherever possible, researchers must adopt procedures and techniques that avoid the use of animals. Where this is not possible, the research should be designed so that;

-The least sentient species with the appropriate physiology is used.
-The number of animals used is the minimum sufficient to provide adequate statistical power to answer the questions posed.
-The severity of procedures performed on animals is kept to a minimum. Experiments should be kept as short as possible. Appropriate anaesthesia, analgesia and humane endpoints should be used to minimise any pain and suffering.

The provisions of the Animals (Scientific Procedures) Act 1986, and any amendments, must be observed and all necessary licences must have been received before any work requiring approval takes place.

Please see "RGC 5 Changes in Research Project" in the event of any proposal to change the arrangements for use of animals in a Research project.

RGC 2.3 Medical and Health Research

The Research Organisation is responsible for managing and monitoring the conduct of medical and health research in a manner consistent with the Department of Health's Research Governance Framework for Health and Social Care (or equivalent). There must be effective and verifiable systems in place for managing research quality, progress and the safety and well-being of patients and other research participants. These systems must promote and maintain the relevant codes of practice and all relevant statutory review, authorisation and reporting requirements.

Research involving human participants or data within the social sciences that falls outside the Department of Health's Research Governance Framework must meet the provisions and guidelines of the ESRC's Research Ethics Framework. While this research may involve patients, NHS staff or organisations, it is defined as research that poses no clinical risk or harm to those who are the subjects of research. Research Organisations must ensure that appropriate arrangements are in place for independent ethics review of social science research that meets local research ethics committee standards.

Significant developments must be assessed as the research proceeds, especially those that affect safety and well-being, which should be reported to the appropriate authorities and to the Research Council. The Research Organisation must take appropriate and timely action when significant problems are identified. This may include temporarily suspending or terminating the research.

The Research Organisation is responsible for managing and monitoring statutory requirements for which it accepts responsibility, for example, in relation to legislation on clinical trials, use of human organs, tissues and data.

Guidance by the MRC on the conduct of medical research, and by ESRC on the conduct of social science research, provided on behalf of all Research Councils, must be observed.

RGC 2.4 Health and Safety

The Research Organisation is responsible for ensuring that a safe working environment is provided for all individuals associated with a research project. Its approach and policy on health and safety matters must meet all regulatory and legislative requirements and be consistent with best practice recommended by the Health & Safety Executive.

Appropriate care must be taken where researchers are working off-site. The Research Organisation must satisfy itself that all reasonable health and safety factors are addressed.

The Research Councils reserve the right to require the Research Organisation to undertake a safety risk assessment in individual cases where health and safety is an issue, and to monitor and audit the actual arrangements made.

RGC 2.5 Misconduct and Conflicts of Interest

The Research Organisation is required to have in place procedures for governing good research practice, and for investigating and reporting unacceptable research conduct, that meet the requirements set out in the Concordat to Support Research Integrity (2012) http://www.universitiesuk.ac.uk/highereducation/Pages/Theconcordattosupportresearchintegrity.aspx and the Research Councils' Code of Conduct and Policy on the Governance of Good Research Conduct.

The Research Organisation must ensure that potential conflicts of interest in research are declared and subsequently managed.

Guidance on providing declarations of interest can be found at
http://www.rcuk.ac.uk/documents/documents/declarationofinterests-applicants-pdf/

RGC 3 Use of Funds

Subject to the following conditions, grant funds may be used, without reference to the Research Council, in such a manner as to

best carry out the research.

Grant funds include a provision for inflation based on the GDP Deflators published by HM Government.

The value of the grant may be varied by the Research Council during the lifetime of the grant in accordance with the deflators or to take into account any other Government decisions affecting the funding available to the Research Councils. Grant funds are provided for a specific research project. Under no circumstances may Directly Incurred and Exceptions funds be used to meet costs on any other grant or activity.

Directly Incurred and Exceptions funds cannot be used to meet the costs of an activity that will fall beyond the actual end date of the grant, e.g. when travel falls after the end of the grant, the costs cannot be charged to the grant even if the tickets, etc. can be purchased in advance.

The Research Councils require public funds to be deployed with due consideration to value for money across all activities.

All travel claims should evidence value for money as the primary consideration. Consequently, these should only include travel by standard class by train and economy class by air for flights. Any exception should be clearly justified and approved within the terms of the RO policy.

See "RGC 11 Equipment" for further information on use of funds for equipment specifically.

RGC 4 Starting Procedures

The process for activating a grant consists of two separate stages. The Research Organisation must formally accept the grant by completing and returning the Offer Acceptance within 10 working days of the offer letter being issued. Returning the Offer Acceptance will result in the Start Confirmation and the Payment Schedule being issued.

The Start Confirmation must be submitted within 42 (calendar) days of the research/training starting and the start date shown on the start confirmation will be regarded as the start date of the grant. The start of the grant may be delayed by up to 3 months from the start date shown in the offer letter, the duration of the grant remaining unchanged. The grant may lapse if it is not started within this period.

The start of the grant may precede the start date shown in the offer letter, but must not be earlier than the date of the offer letter itself.

The start of the grant should be defined as follows:

-For research grants with DI staff; the date on which the first DI staff supported by the grant start work.
-For research grants with DI staff, but where it is intended that staff should not be in post at the start of the grant; the date on which expenditure on any other DI or DA (excluding estates) heading first occurs;
- For research grants without DI staff: the date on which any DI or DA (excluding estates) expenditure first occurs.

Grants may not be started in any other way without prior approval from the Research Council.

Expenditure may be incurred prior to the start of the grant and be subsequently charged to the grant, provided that it does not precede the date of the offer letter.

RGC 5 Changes in Research Project

The Research Council must be consulted in the event of any major change in the proposed research, including failure to gain access to research facilities and services, or to gain ethical committee approval for the research, particularly those which make it unlikely that the objectives of the research can be achieved.

In addition, for research involving the use of animals or human participation, any substantive changes from the experimental design endorsed by the awarding Board or Panel that might impact on the ethical characteristics of the award must be authorised by the Research Council. Such changes would include, but may not be limited to, the use of different animal species and/or the experimental design or clinical protocol.

If appropriate, revised proposals may be required. The Research Council reserves the right to make a new grant in place of the existing grant, or to revise, retain or terminate the existing grant.

It is the responsibility of the Research Organisation to manage the resources on the grant, including the staff, and the Research Council need not be consulted if staffing levels on the grant are changed. However, a proportionate reduction should be made in the value of Estates, Indirect Costs and Infrastructure Technicians claimed by the Research Organisation in the following circumstances:

- a post that attracts these costs is not filled.
- a staff member who attracts these costs leaves more than six months before the end of the period for which the post was funded and is either not replaced, or is replaced by a category of staff that does not attract the costs e.g. project student or technician.

RGC 6 Transfers of Funds between Fund Headings

Transfers of funds between fund headings are permitted only within and between Directly Incurred costs and Exceptions, excluding

equipment.

Funds may only be transferred into studentship stipend or fees to supplement an existing studentship post on the grant. They may not be transferred to create new posts without prior approval from the Council. Transfers will be at the rate applicable for the heading, as set out in the award letter.

Funds can only be transferred and used to meet the cost of activity or activities that meet the agreed aims and objectives of the project. While approval does not need to be sought from the Research Council for transfer of funds, the Research Councils reserve the right to query any expenditure outlined in the Final Expenditure Statement, which has not been incurred in line with the Grant Terms and Conditions.

See "RGC 11 Equipment" for further information on the transfer of funds for equipment specifically.

RGC 7 Extensions

For Research Grants: After a research grant has started, the duration may be extended at no additional cost by an overall total of up to 12 months, subject to prior written approval. Extensions will be allowed where they are necessary to enable work to be completed following delays due to:

- breaks or delays in the appointment of staff;
- maternity, paternity, adoption, shared parental or paid sick leave;
- extended jury service; or
- changes from full-time to part-time working.

In the case of other, exceptional, circumstances, the duration may be extended, at the discretion of the Research Council.

Extensions will be limited to the additional time needed to complete the research. Any request for an extension should therefore state the reasons for the delay and explain how the extra time requested will enable the remaining work to be completed.

Fellowship Grants: After a fellowship grant has started, the duration may be extended to cover maternity leave, paternity leave, adoption leave, shared parental leave, extended jury service or paid sick leave for a Research Fellow in line with the terms and conditions of the fellow's employment. Otherwise, the conditions for extending Fellowship grants are the same as apply to research grants.

Requests for extensions should be made via the Grant Maintenance facility in Je-S once the required duration is known and before the grant ends.

RGC 8 Staff

The Research Organisation must assume full responsibility for staff funded from the grant and, in consequence, accept all duties owed to and responsibilities for these staff, including, without limitation, their terms and conditions of employment and their training and supervision, arising from the employer/employee relationship.

The Research Organisation must provide research staff with a statement, at the outset of their employment, setting out the provisions for career management and development, including personal skills training, and ensure that they have access to appropriate training opportunities.

Provided it is related to the research project on which they are currently working, Research staff and Research Fellows may, during normal working hours, undertake teaching and demonstrating work, including associated training, preparatory, marking and examination duties, for up to an average of 6 hours a week (pro rata for part-time staff) calculated over the period that they are supported on the grant.

RGC 9 Maternity, Paternity, Adoption and Parental Leave

The Research Organisation will be compensated at the end of the grant to cover any additional net costs that cannot be met within the cash limit of paid parental leave (ie maternity, paternity and adoption leave) for staff within the Directly Incurred and Exceptions fund headings (excluding the principal and co-investigators, unless they are also research fellows or research assistants funded by the grant) if they fulfil the relevant qualifying conditions of the employing Research Organisation. The net cost is the amount paid to the individual less the amount the Research Organisation can recover for Statutory Maternity Pay and Statutory Adoption Pay from HMRC.

Parental leave pay is payable by the Research Council only for directly incurred staff that are funded for 100% of their contracted time on the grant (apart from staff acting as principal or co-investigators unless they are also research fellows or research assistants funded by the grant).

Grant funds, within the announced cash limit, may be used to meet the costs of making a substitute appointment and/or extending the grant to cover a period of parental leave for staff within the directly incurred and exceptions fund headings (excluding the principal and co-investigators, unless they are also research fellows or research assistants funded by the grant). The duration of a grant will be extended only if the period can be accommodated within the maximum period allowed for extensions. Directly Allocated and Indirect funds will not be increased as a result of such extensions.

Research Grants: Research Grant funds may be used to meet the costs of paid parental leave only to the extent that it is taken

during the original period of the grant. The Research Organisation will be responsible for any liability for parental leave pay for staff supported by the grant outside the original period of the grant. If, for example, the original end date of a grant falls while a member of research staff is part-way through her maternity leave, the Research Organisation will be responsible for that part of the maternity leave which is taken after the original end date.

Fellowship Grants: Fellows are entitled to take parental leave in accordance with the terms and conditions of the fellow's employment. If requested, consideration will be given to allowing a fellowship grant to be placed in abeyance during the absence of the Research Fellow for parental leave, and the period of the fellowship extended by the period of leave. Consideration will be given to requests to continue the fellowship on a flexible or part--time basis to allow the Research Fellow to meet caring responsibilities.

RGC 10 Sick Leave

The Research Organisation will be compensated at the end of the grant to cover any additional net costs, that cannot be met within the cash limit, of paid sick leave for staff within the Directly Incurred and Exceptions fund headings (excluding the principal and co-investigators, unless they are also Research Fellows or Research Assistants funded by the grant) who fulfil the qualifying conditions of the Research Organisation. The net cost is the amount paid to the individual less the amount the Research Organisation can recover from HMRC.

Sick pay is payable by the Research Council only for directly incurred staff that are funded for 100% of their contracted time on the grant (apart from staff acting as principal or co-investigators unless they are also research fellows or research assistants funded by the grant).

Grant funds, within the announced cash limit, may be used to meet the approved costs of making a substitute appointment and/or extending the grant to cover a period of sick leave for staff within the directly incurred and exceptions fund headings (excluding the principal and co-investigators, unless they are also research fellows or research assistants funded by the grant). The duration of a grant will be extended only if the period can be accommodated within the maximum period allowed for extensions. Directly Allocated and Indirect funds will not be increased as a result of such extensions.

Research Grants: Research Grant funds may be used to meet the costs of paid sick leave only to the extent that it is taken during the original period of the grant. The Research Organisation will be responsible for any liability for sick leave pay for staff supported by the grant outside the original period of the grant.

Where there is a continuous period of sick leave in excess of 3 months, the Research Organisation may apply to the Research Council to discuss the possibility of a substitute appointment to safeguard progress on the project. Where a Research Assistant has been on sick leave in excess of 3 months the Research Organisation must comply with all their obligations to consider reasonable adjustments before making a substitute appointment. Where a Research Assistant has been on sick leave for an aggregate (not necessarily continuous) period in excess of 3 months, where this is due to a single condition or a series of related conditions, the Research Organisation may request an extension to the duration of the project.

Fellowship Grants: Fellows are entitled to take sick leave in accordance with the Research Organisation's terms and conditions. If requested, consideration will be given to allowing a fellowship grant to be placed in abeyance during the absence of the Research Fellow due to sick leave, and the period of the fellowship extended by the period of sick leave. The additional salary costs for the fellow (pro rata to their percentage FTE on the fellowship) should be claimed, as necessary, at the end of the extended period.

RGC 11 Equipment

RGC 11.1 Procurement of Equipment

The procurement of equipment, consumables and services, including maintenance, must comply with all relevant national and EU legislation and the Research Organisation's own financial policy and procedures. Accepted procurement best practice in the higher education sector must be observed. For all equipment and services where the contract value is more than £25,000, excluding VAT, professionally qualified procurement staff must be consulted before the procurement process begins, and, where appropriate, at the market research stage, and must approve the order/contract before it is placed with a supplier.

RGC 11.2 Ownership of Equipment

Equipment purchased from grant funds is primarily for use on the research project for which the research grant was awarded, and belongs to the Research Organisation. In certain circumstances the Research Council may wish to retain ownership throughout the period of the grant and possibly beyond. In such cases, the grant will be subject to an additional condition.

The Research Council must be informed if, during the life of the research grant, the need for the equipment diminishes substantially or it is not used for the purpose for which it was funded. The Research Council reserves the right to determine the disposal of such equipment and to claim the proceeds of any sale. Any proposal to transfer ownership of the equipment during the period of the grant is subject to prior approval by the Research Council. After the research project has ended, the Research Organisation is free to use the equipment without reference to the Research Council, but it is nevertheless expected to maintain it for research purposes as long as is practicable.

RGC 11.3 Use of Equipment

Where there is spare capacity in the use of the equipment, the Research Council expects this to be made available to other users. Priority should be given to research supported by any of the Research Councils and to Research Council-funded students.

RGC 11.4 Use of Equipment Funds

Any proposal to purchase an item of equipment in the last 6 months of the grant is subject to prior written approval by the Research Council. The Research Council will wish to be assured that the item of equipment is essential to the research.

Equipment funding is ring-fenced and transfers into or out of the equipment headings, whether under Directly Incurred or Exceptions, is not permitted.

RGC 11.5 Equipment.Data

In line with the recommendation made in the "Efficiency, effectiveness and value for money" report (http://www.universitiesuk.ac.uk/policy-and-analysis/reports/Documents/2015/efficiency-effectiveness-summary.pdf) , all new equipment purchased over £138,000 (£115,000 ex VAT) using public funding sources should be registered on the equipment.data.ac.uk national database to be discoverable and enable greater sharing.

RGC 12 Transfer of a Grant to another Research Organisation

The Research Organisation must send a request via the Grant Maintenance facility in Je-S if the Grant Holder intends to transfer to another organisation. If this organisation is eligible to hold grants, and is able to provide a suitable environment to enable the project to be successfully completed, the expectation is that the grant would be transferred with the Grant Holder. Written agreement to this is required from both the relinquishing and receiving organisations; this will normally be triggered automatically by the initial request to Je-S.

The Research Council will wish to be assured that satisfactory arrangements have been agreed that will enable the project to be undertaken, or to continue, in accordance with its research objectives. If suitable arrangements cannot be agreed, the Research Council will consider withdrawing its support or terminating the grant.

Where there is a basis for continuing involvement by the relinquishing organisation, agreement should be reached between both organisations on the apportionment of work and the distribution of related funding. Grants will not be re--costed following transfer. The unspent balance of Directly Incurred and Exceptions costs will be transferred to the receiving Research Organisation. In the case of Directly Allocated and Indirect costs, a pro rata share, based on the time elapsed on the grant at the point of transfer, will be transferred to the receiving research organisation. The receiving organisation will be required to confirm, by return of an offer acceptance, that it will provide any additional resources needed to complete the project.

RGC 13 Change of Grant Holder

Research Grants: The Research Organisation must consult the Research Council via the Grant Maintenance facility in Je-S if it is proposed to change the Grant Holder, for example, following retirement or resignation. Where the Grant Holder is transferring to another organisation eligible to hold a grant, the provisions of "RGC 12 Transfer of a Grant to another Research Organisation" will apply. In other circumstances, the Research Organisation may nominate a replacement Grant Holder. The Research Council will wish to be assured that the replacement meets the eligibility criteria and has the expertise and experience to lead the project to a successful conclusion, in accordance with its research objectives.

Fellowship Grants: A fellowship grant is awarded on the basis of a named individual's suitability to undertake and benefit from the period of research therefore changes to the Grant Holder are not permitted. The resignation of the Research Fellow, or the termination of their employment, constitutes the end of the grant for the purpose of submitting a final report and the Council's financial liabilities.

RGC 14 Annual Statement

The Research Organisation may be sent a statement to return each year showing payments made by the Research Council during the previous financial year for all the grants it holds. Where a statement is required, the Research Organisation must certify, by returning the statement, that:

- Expenditure has been incurred in accordance with the grant conditions, and
- Those grants shown as current are continuing.

No further payments will be made until the annual statement has been received and accepted by the Research Council.

RGC 15 Expenditure Statements

The Research Organisation is accountable for funds dispersed and must complete and return an expenditure statement within 3 months of the end date of a grant. If it is not returned within this time then the terms stated in "RGC 18.2 Organisation Sanctions" will apply. Once an expenditure statement has been received and the expenditure incurred has been reconciled against payments made, it will be considered as final. Any unspent funds will be recovered.

Expenditure shown in the Directly Incurred and Exceptions headings must show the actual expenditure incurred by the project. Settlement by the Research Council will reflect the proportion of fEC stated in the award letter applied to actual expenditure, within the cash limit.

For the Directly Allocated and Indirect Costs headings, the Research Council will pay the amount shown as spent, within the cash

limit, provided that the grant ran its full course. Where a grant is terminated more than 6 months before the planned end date, a pro rata share will be paid. Where a grant terminates within 6 months of the planned end date, estates and Indirect Costs will be paid in full, but Investigators' costs and Other Directly Allocated Costs will be paid pro rata.

Costs arising from parental or sick leave should be identified in the Absence heading of the statement.

The Research Council reserves the right to require the Research Organisation to complete and submit a statement of expenditure at any time during the course of a grant, or to provide supplementary information in support of an interim or final expenditure statement.

If there are exceptional reasons that will prevent submission of the expenditure statement within the period allowed, a written request may be made via the Grant Maintenance facility in JeS, before the due date passes, for the submission period to be extended.

RGC 16 Disclosure and Inspection

The Research Council reserves the right to have reasonable access to inspect the records and financial procedures associated with grants or to appoint any other body or individual for the purpose of such inspection. This includes expenditure by third parties. Research Councils shall be entitled to request and/or have access to any financial records and reports that are deemed appropriate to demonstrate the regularity and propriety of expenditure, including but not limited to:
o Annual report & accounts
o External audit management letter
o ISA260 - Communication with those charged with governance
o Related internal audit reports

The Research Organisation must report to the Research Council:

-Any investigations (and their outcomes) into research misconduct associated with the grant at the stage that it is decided to undertake an informal inquiry; and
-On request provide information on:
o its management of research integrity and ethics as described at: http://www.rcuk.ac.uk/funding/researchintegrity/
o Details of any retractions or withdrawal of submissions/publications
-Any allegations, proven or not, of any cases of fraud.

The Research Organisation must, if required by the Research Council, provide a statement of account for the grant, independently examined by an auditor who is a member of a recognised professional body, certifying that the expenditure has been incurred in accordance with the research grant terms and conditions.

Research Councils will undertake periodic reviews of Research Organisations within the Funding Assurance Programme to seek assurance that grants are managed in accordance with the terms and conditions under which they are awarded.

RGC 17 Reporting on the conduct and results of research

The Research Councils use an online system to collect information during the lifetime of the grant and for some years afterwards on the outputs and outcomes of research, and provide guidance on the timing and scope of reporting that is required. The Research Organisation must ensure that the system is used in accordance with the guidance provided.

Exceptionally, the Research Council may require a separate final report on the conduct and outcome of the project. If so, it must be submitted by the Research Organisation within three months of the end of the grant, on the form provided. No further application from a Grant Holder will be considered while a final report is overdue.

RGC 18 Sanctions

The Research Councils reserve the right to impose financial sanctions and/or additional measures where they identify areas of non-compliance with these terms and conditions of grant.

RGC 18.1 Contact Sanctions

If outputs and outcomes are not reported as directed the Research Councils collectively will not consider further proposals where the grant holder is named as the Principal or Co Investigator. In addition the Research Councils will suspend payments for the associated grant.

RGC 18.2 Organisation Sanctions

If the final report or the financial expenditure statement is not received within 3 months of the end date of the grant, the Research Council will recover 20% of expenditure incurred on the grant. All payments will be recovered if the report or statement is not received within 6 months of the end of the grant. Research Organisations may appeal against a sanction, but must do so within 60 days of the pay run in which the sanction was imposed.

The Research Council shall be entitled to suspend payments or recover funds on grants in the event that the Research Organisation does not comply with the terms and conditions of grant.

In relation to the current Quality Assurance and validation project for TRAC implementation in universities, the Research Councils reserve the right to apply sanctions of 75% of the non-compliant rate where an institution is found to be using rates which are

materially inaccurate (>10% variance on any single rate). These sanctions would only apply to future applications although Councils may exercise a higher sanction where there has been evidence of significant overpayments to research organisation based on inaccurate rates.

RGC 19 Public Engagement

It is the responsibility of the Research Organisation and the Grant Holder and Co--Investigators to communicate the research to the public at both local and national level, and to raise awareness of the role of science and research in any related issues of public interest. Special schemes exist in some Research Councils providing additional support for these activities.

RGC 20 Exploitation and Impact

It is the responsibility of the Research Organisation, and all engaged in the research, to make every reasonable effort to ensure that the intellectual assets obtained in the course of the research, whether protected by intellectual property rights or not, are used to the benefit of society and the economy. Research outcomes should be disseminated to both research and more widespread audiences, for example to inform potential users and beneficiaries of the research.

Unless stated otherwise, the ownership of all intellectual assets, including intellectual property, and responsibility for their application, rests with the organisation that generates them.

Where the grant is associated with more than one research organisation and/or other project partners, the basis of collaboration between the organisations, including ownership of intellectual property and rights to exploitation, is expected to be set out in a formal collaboration agreement. It is the responsibility of the Research Organisation to put such an agreement in place before the research begins. The terms of collaboration agreements must not conflict with the Research Councils' terms and conditions.

Arrangements for collaboration and/or exploitation must not prevent the future progression of research and the dissemination of research results in accordance with academic custom and practice. A temporary delay in publication is acceptable in order to allow commercial and collaborative arrangements to be established.

The Research Council may, in individual cases, reserve the right to retain ownership of intellectual assets, including intellectual property (or assign it to a third party under an exploitation agreement) and to arrange for it to be exploited for the national benefit and that of the Research Organisation involved. This right, if exercised, will be set out in an additional grant condition.

There should be suitable recognition and reward to researchers who undertake activities that deliver benefit through the application of research outcomes. The Research Organisation must ensure that all those associated with the research are aware of, and accept, these arrangements.

RGC 21 Research Monitoring and Evaluation

While it is the responsibility of the Research Organisation to manage the research, the Research Council reserves the right to call for periodic information on progress or to visit the project team. The Grant Holder may also be asked to attend meetings to exchange information and ideas with others undertaking research in the same or similar fields.

The Grant Holder must make all reasonable efforts, if so invited, to respond to requests for information or to attend events or activities organised by the Research Council concerning the research undertaken. Such events may be held after a grant has finished.

RGC 22 Publication and Acknowledgement of Support

The Grant Holder should, subject to the procedures laid down by the Research Organisation, publish the results of the research in accordance with normal academic practice and the RCUK policy on open access http://www.rcuk.ac.uk/documents/documents/rcukopenaccesspolicy-pdf/

Publications and other forms of media communication, including media appearances, press releases and conferences, must acknowledge the support received from the Research Council (or Councils, in the case of grants funded by more than one) quoting the grant reference number if appropriate. Journal publications should acknowledge the funding source using the standard format agreed by funders and publishers and detailed in the additional information accompanying this grant.

RGC 23 Disclaimer

The Research Councils accept no liability, financial or otherwise, for expenditure or liability arising from the research funded by the grant, except as set out in these terms and conditions, or otherwise agreed in writing.

Where studies are carried out in an NHS Trust, the Trust has a duty of care to its patients. The Research Council does not accept liability for any failure in the Trust's duty of care, or any negligence on the part of its employees.

The Research Councils reserve the right to terminate the grant at any time, subject to reasonable notice and to any payment that may be necessary to cover outstanding and unavoidable commitments.

Further to "RGC 3 Use of Funds", the Research Councils reserve the right to amend the payment profile at their discretion. The Research Organisation will be advised, in advance, of any such a change. Changes to payment profiles may affect the overall value of the grant.

If a grant is terminated or reduced in value, no liability for payment or redundancy or any other compensatory payment for the

dismissal of staff funded by the grant will be accepted, but, subject to the provisions of "RGC 15 Expenditure Statements", negotiations will be held with regard to other contractual commitments and concerning the disposal of assets acquired under the research grant.

RGC 24 Status

These terms and conditions will be governed by the laws of England and Wales; all matters relating to the terms and conditions will be subject to the exclusive jurisdiction of the courts of England and Wales.

If any provision of these terms and conditions is found by a court or other legitimate body to be illegal, invalid or unreasonable, it will not affect the remaining terms and conditions which will continue in force.

These terms and conditions, together with any additional conditions set out in the grant; contain the whole agreement between the Research Council and the Research Organisation in relation to the stated research grant. The Research Council and the Research Organisation do not intend that any of these terms and conditions should be enforceable by any third party.

ADDITIONAL GRANT CONDITION: ASSET TRANSFER TO UKRI

This Additional Condition will be incorporated into the Standard Grant Terms and Conditions when they are next updated in 2018.

1 The parties acknowledge that United Kingdom Research and Innovation will be established as a body corporate in accordance with the Higher Education and Research Act 2017 and that the property, rights and liabilities of the awarding Research Council will be acquired by United Kingdom Research and Innovation in accordance with the provisions of that Act.

2 The parties agree that on and with effect from the date on which the UKRI Property Transfer Scheme becomes effective in accordance with its terms (the Scheme Effective Date):

2.1 all rights and benefits of the awarding Research Council arising out of or in connection with this agreement; and

2.2 all obligations and liabilities of the awarding Research Council arising out of or in connection with this agreement, shall (in each case) be transferred to, and assumed by, United Kingdom Research and Innovation on the basis set out in the UKRI Property Transfer Scheme.

3 The grant awardee undertakes that, at any time and from time to time on or after the Scheme Effective Date, it will execute such documents and take such other action as the awarding Research Council may reasonably request in order to implement and give effect to (i) the transfer of the rights, benefits, obligations and liabilities of the awarding Research Council arising out of or in connection with this agreement to United Kingdom Research and Innovation; and (ii) the release and discharge of the awarding Research Council in respect of such obligations and liabilities.

4 In this clause UKRI Property Transfer Scheme means a property transfer scheme made by the Secretary of State in accordance with the provisions of Schedule 10 to the Higher Education and Research Act 2017 and references to transfer and transferred shall be construed as references to assignment, novation or to the steps which are necessary to give effect to the arrangements contemplated by this clause.

MRC Additional Terms and Conditions

The MRC additional terms and conditions of funding supplement those of RCUK. These conditions set out operational, legislative and ethical requirements relating to medical research. The MRC reserves the right to vary these additional terms and conditions.

Research organisations and award holders (Award Holders are all MRC Grant Holders and recipients of MRC Unit and Institute funding programme leaders) have absolute responsibility for ensuring all required licenses, approvals, permissions and consent are in place before any research is undertaken and that these are followed.

MRC reserves the right to audit at any time without prior notice:

- That required licenses, approvals, permissions and consent are in place, or were in place when the activity occurred.

- Compliance with the terms and conditions set out here.

AC1 Responsibilities of the Research Organisation: Clinicians

The research organisation is responsible for ensuring all clinicians supported by MRC funding are aware they are individually responsible for maintaining appropriate professional indemnity insurance. This should be with a professional defence organisation for any activities not covered by NHS indemnity arrangements or by additional provision made by the research organisation. MRC will not meet the costs of such cover.

The research organisation is responsible for ensuring any honorary clinical contracts required by clinical staff have been obtained prior to the start of the research.

The MRC expects the research organisations to abide by the 'UK clinical academic training in medicine and dentistry: principles and obligations' (www.mrc.ac.uk/documents/pdf/clinical-principles-and-obligations-report/).

AC2 Clinical Responsibilities

Research staff supported full-time by an MRC grant may work up to six hours a week during normal work hours on NHS clinical sessions. Exceptions are made for surgeons, who may undertake up to three clinical sessions a week, and fellows undertaking patient-oriented research, who may undertake up to four clinical sessions a week.

All holders of clinical fellowship grants (Clinical Research Training Fellowships, Clinician Scientist Awards or Senior Clinical Fellowships) may choose to spend up to 20% (on average over the lifetime of the grant) of their normal working hours on NHS clinical sessions, teaching and demonstrating, or research activities beyond the scope of their fellowship. This is not in addition to the six hours per week all research staff supported full-time by an MRC grant or fellowship may undertake under RGC 8 of the RCUK Terms and Conditions of Research Council fEC Grants (www.rcuk.ac.uk/funding/grantstcs/). Fellowship Grant holders who specified a time commitment of more than 20% to these duties in their proposal (for example, due to specialty demands or the patient-oriented nature of the research) may choose to spend up to the time specified in their proposal on these activities.

AC3 Publicity for MRC-Funded Research

All research results and achievements should be communicated to the MRC Press Office (press.office@headoffice.mrc.ac.uk) before publication.

Award holders must inform the MRC Press Office as soon as a paper presenting MRC-funded research is accepted for publication. The MRC reserves the right to lead on publicity when the MRC is the majority funder. The MRC Press Office must be notified at least 5 working days in advance of any publicity arising from MRC funding, and any press releases referencing the MRC must be approved by the MRC Press Office before it is released to the media.

AC4 Use of Animals

The MRC supports the principles of the 3Rs (Replacement, Reduction and Refinement). Research organisations and award holders are expected to abide by the core principles set out in the cross-funder guidance 'Responsibility in the use of animals in bioscience research: Expectations of the major research councils and charitable funding bodies' (available at www.nc3rs.org.uk) and RGC 2.2 of the RCUK Terms and Conditions (www.rcuk.ac.uk/funding/grantstcs/).

The provisions of the Animals (Scientific Procedures) Act 1986 must be observed. All MRC awards are made on the absolute condition that no work which is controlled by the act will begin until the necessary licences have been obtained from the Home Office. Any recommendations arising from the MRC peer review process with regards to animal use must be followed.

When animals are purchased from commercial suppliers, UK suppliers should be used wherever possible, to minimise the risk of suffering during transport.

All research involving non-human primates must comply with the NC3Rs Guidelines: Primate accommodation, care and use (available at www.nc3rs.org.uk).

Researchers should ensure that they report animal-based studies in accordance with the ARRIVE guidelines (www.nc3rs.org.uk/ARRIVE) as far as possible, taking into account the specific editorial policies of the journal concerned.

Any new procedure likely to replace the use of animals in research or testing, reduce the numbers used or refine animal use must be reported to the MRC and disseminated through the usual channels to all those who might make use of it.

MRC is a public body legally obliged to provide information on its work to parliament and to the public, and is committed to improving transparency in public communications on animal use. MRC will make public information about the animal experiments it funds when needed (for example as anonymous examples, or in response to direct queries). MRC will resist all requests for information that might lead to the identification of places or individuals, except with the express permission of the individuals concerned.

AC5 Mouse Strains

MRC supports a central repository of mouse strains - the MRC mouse Frozen Embryo and Sperm Archive (FESA) at the Mammalian Genetics Unit, Harwell. Award holders are expected to contact FESA to highlight mouse strains engineered, or characterised using MRC funds, and are encouraged to deposit these strains with the archive.

Depositors retain ownership of strains and there is currently no charge for depositing strains to make them freely available to the academic community.

FESA aims to ensure that valuable mouse strains are safeguarded, that the need to maintain colonies of live mice for long periods of time is reduced, and that the significant investment in engineering strains is capitalised upon fully. MRC award holders planning mouse research should contact FESA at the earliest opportunity.

For help with the requirements of AC6-AC13 please contact MRC Regulatory Support Centre: www.mrc.ac.uk/research/facilities-and-resources-for-researchers/regulatory-support-centre/

AC6 Health Departments' Research Governance Frameworks

Research involving NHS patients, their organs, tissues or data which falls within the scope of the UK Health Departments' Research Governance Frameworks (RGF, www.hra.nhs.uk/resources/research-legislation-and-governance/research-governance-

frameworks/) must comply with MRC policy on the health departments research governance frameworks (www.mrc.ac.uk/research/policies-and-guidance-for-researchers/clinical-research-governance/health-departments-research-governance/).

MRC requires research organisations to ensure sponsorship responsibilities are clearly identified, the research undertaken complies with the requirements of the employing organisation set out in the RGF, and that agreements and systems are in place with NHS Trusts and other partner organisations, including commercial organisations, to comply with the RGF. Systematic documentation of key decisions and approvals, particularly in relation to work with patients, their organs, tissues and data is crucial.

AC7 Human Participants in Research

MRC expects all research involving human participants to be undertaken in accordance with its policies and guidance available from www.mrc.ac.uk/research/policies-and-guidance-for-researchers/#ethics.These include:

- Good Research Practice (2012);
- Medical research involving adults who cannot consent (2007);
- Medical Research Involving Children (2004);
- Human Tissue and Biological Samples for Use in Research (2014);
- Personal Information in Medical Research (2000)

Research organisations and award holders have absolute responsibility for ensuring that investigations being undertaken within NHS premises, nursing or residential homes or NHS service establishments, schools, or any other organisations, do not take place without the explicit approval of the appropriate authority in advance.

Payments to healthy volunteers participating in clinical research are allowable, provided that the payment is for expense, time and inconvenience and is not at a level which would induce people to take part in studies against their better judgement. Further guidance on payments and incentives in research can be found at www.hra.nhs.uk/documents/2014/05/hra-guidance-payments-incentives-research-v1-0-final-2014-05-21.pdf

Independent Research Ethics Committee approval is required for research that involves human participants (whether patients or healthy volunteers) or records. In the case of research involving NHS patients, premises or records, this will be a NHS Research Ethics Committee (REC). Such approval is also required for certain studies of human tissues. Further guidance on when NHS REC approval is required can be found at www.hra-decisiontools.org.uk/ethics/
In England and Wales research involving individual patient data, where the patient's consent will not be obtained, is covered by "Section 251" of The National Health Service Act 2006, and requires additional approval via the Health Research Authority's Confidentiality Advisory Group (www.hra.nhs.uk/about-the-hra/our-committees/section-251/). In Scotland, decisions on disclosure of identifiable patient information are made by Caldicott Guardians (see www.informationgovernance.scot.nhs.uk/ for further details).

In the case of social science research, the MRC recommends that award holders follow the ESRC Framework for Research Ethics (revised 2015, www.esrc.ac.uk/files/funding/guidance-for-applicants/esrc-framework-for-research-ethics-2015/) which highlights the responsibility of the research organisation for ensuring that the research is subject to appropriate ethics review. In some cases this review is required by an NHS REC, for further guidance please see www.hra.nhs.uk/research-community/

MRC requires the award holder to notify MRC if amendments required by a regulator or a REC will substantially affect the research question, methodology or cost previously approved.

Any serious incident arising in the course of research that has been approved by a REC should be reported immediately to the MRC, as well as to the REC. The research must be suspended until the REC has decided whether it may be continued or should be abandoned.

Research involving human participants in developing societies presents specific ethical challenges and the MRC guidelines Research Involving Human Participants in Developing Societies (www.mrc.ac.uk/publications/browse/research-involving-human-participants-in-developing-societies/) must be followed.

AC8 Clinical Trials

When research involves MRC-funded clinical trials, award holders must act in accordance with MRC policy on UK clinical trials regulations (www.mrc.ac.uk/research/policies-and-guidance-for-researchers/clinical-research-governance/clinical-trials-regulations/) in relation to ethical, sponsorship, reporting, monitoring and publication requirements.

- An independent Trial Steering Committee and Data Monitoring and Ethics Committee must be set up to oversee the conduct of the trial, with an MRC representative acting as an observer.

- MRC-funded trials must be registered with an International Standardised Randomised Control Trial Number (ISRCTN) on the ISRCTN Registry (www.isrctn.com). The unique identification number must be used in publications and provided to MRC by adding it to Researchfish within a year of the trial starting. Failure to provide this number will result in suspension of funding.

- Results of MRC-funded trials (whether positive or negative) must be published without unreasonable delay following the conclusion of the study (generally within a year of completion). Results should be reported in accordance with the recommendations in the CONSORT statement (www.consort-statement.org/). Before results are published they must be discussed by the Trial Steering Committee.

- Any contribution to an MRC-funded trial by another body, such as a pharmaceutical company (donation of drugs etc.), must be the

subject of a collaboration agreement between the parties (see AC20).

AC9 Data Sharing

Award holders must comply with the MRC policy on research data sharing (www.mrc.ac.uk/documents/pdf/mrc-data-sharing-policy/) along with the MRC policy on sharing of research data from population and patient studies (www.mrc.ac.uk/publications/browse/mrc-policy-and-guidance-on-sharing-of-research-data-from-population-and-patient-studies/).

AC10 Human Fertilisation

When research involves the use of human gametes, embryos or human admixed embryos researchers must act in accordance with the Human Fertilisation and Embryology Act 1990 as amended in 2008 and 2015 (the Human Fertilisation and Embryology (Mitochondrial Donation) Regulations). This includes obtaining a research licence to undertake activities covered by the Act. Further information can be obtained from www.hfea.gov.uk/

AC11 Medical Records

When research involves the use of medical records, the award holder must act in accordance with the principles set out in the Data Protection Act 1998 and the NHS requirements to protect patient confidentiality. Advice on these requirements is available from the MRC Regulatory Support Centre.

All researchers handling personal data must have clearly established obligations to maintain confidentiality (eg formalised within policy written by their research organisations or through professional codes of conduct).

All NHS bodies should routinely inform patients that medical information may be used in research statistics, etc., and should give patients who wish to discuss any concerns an opportunity to do this (Section 251 of NHS Act 2006). Identifiable data should not be used in research if a patient has made clear that they do not wish it to be.

AC12 Removal, Use or Storage of Human Tissue

Award holders whose research involves the removal, use or storage of human tissue as specified in the relevant legislation must:

- Comply with the appropriate legislation, ie the Human Tissue Act 2004 and/or the Human Tissue (Scotland) Act 2006;
- Follow the relevant standards and Codes of Practice issued by the Human Tissue Authority (HTA) (the MRC Regulatory Support Centre (www.mrc.ac.uk/research/facilities-and-resources-for-researchers/regulatory-support-centre/) has summarised these);
- Follow the MRC guidance detailed in Human Tissue and Biological Samples for Use in medical Research (2014,www.mrc.ac.uk/publications/browse/human-tissue-and-biological-samples-for-use-in-research/).

Where research involves the use of human tissues and cells to treat patients (human application), award holders must also:

- Comply with the Human Tissue (Quality and Safety for Human Application) Regulations 2007;
- Work within the applicable regulations and standards as dictated by the Human Tissue Authority, Medicines and Healthcare products Regulatory Agency (MHRA), Human Fertilisation and Embryology Authority and Health Research Authority. The UK Stem Cell Tool Kit (www.sc-toolkit.ac.uk/home.cfm) gives guidance on applicable regulatory routes, and the MHRA Innovation Office (www.gov.uk/government/groups/mhra-innovation-office) provides a regulatory advice service for regenerative medicine.

When research involves the use of human fetal tissue, or non-fetal products of conception (ie amniotic fluids, umbilical cord, placenta or membranes), researchers should follow the guidance set out in relevant Codes of Practice issued by the HTA (in particular see paragraphs 171-175 in the Code of Practice on Consent at www.hta.gov.uk/).

When research involves procedures for the removal of human tissue at post-mortem examination, researchers must also follow guidance issued by the Health Departments and Local Health Authorities.

AC13 Stem Cells

Award holders whose research involves human stem cell lines (both embryonic and adult) must:

- Abide by the UK Code of Practice for the use of Human Stem Cell lines (www.mrc.ac.uk/documents/pdf/code-of-practice-for-the-use-of-human-stem-cell-lines/)

- Ensure that they hold all relevant licenses, accreditations and approvals from, and abide by the Codes of Practice issued by, but not limited to, the Human Fertilisation and Embryology Authority (HFEA; see AC10), the Human Tissue Authority (HTA; see AC12), the Health Research Authority (HRA; for research ethics, gene therapy and confidentiality; see AC6, AC7, AC8), the Medicines and Healthcare products Regulatory Agency (MHRA; see AC6, AC7, AC8), the EU Tissue and Cells Directive (where applicable).

In the case of research involving human embryonic stem cells:

- Deposit a sample of every human embryonic stem cell line derived with MRC funding in the UK Stem Cell Bank; applications to deposit or access banked stem cell lines must be approved by the Steering Committee for the UK Stem Cell Bank and for the Use of Stem Cell Lines (www.mrc.ac.uk/research/policies-and-guidance-for-researchers/uk-stem-cell-bank-steering-committee/).

- Not pass samples of human embryonic stem cell lines to third parties other than those approved by the Steering Committee for the UK Stem Cell Bank and for the Use of Stem Cell Lines and/or the HFEA.

- Not take human embryonic stem cell lines out of the UK unless approved by the Steering Committee for the UK Stem Cell Bank

and for the Use of Stem Cell Lines and/or the HFEA.

- Scientists from overseas wishing to conduct human embryonic stem cell research in the UK as visiting workers must provide a written statement from their home institution, outlining that as the employer of the visiting worker they take on the responsibilities of ensuring their employee works to and complies with the requirements of the UK Governance landscape, set out in the UK Code of Practice.

- Send copies of publications to the UK Stem Cell Bank, and agree that the UK Stem Cell Bank may post summaries of published results on their web site.

- Assist the MRC and the UK Stem Cell Bank, on request, with public engagement activities.

AC14 Use of Radioactive Substances and Neutron Irradiation in Humans

When research requires the administration of radioactive medicinal products (including in vivo neutron activation analysis in humans), researchers must follow the guidance issued by the Administration of Radioactive Substances Advisory Committee (ARSAC, www.gov.uk/government/organisations/administration-of-radioactive-substances-advisory-committee/about) and seek the relevant approval(s) as appropriate.

AC15 Genetic Modification

In accordance with the Genetically Modified Organisms (Contained Use) Regulations 2014, research organisations and individuals undertaking genetic modification must be registered with the Health and Safety Executive (HSE), undertake risk assessment and seek consent where appropriate.

Researchers who carry out genetic modification should be familiar with the legislative requirements and with the Scientific Advisory Committee on Genetic Modification (Contained Use) guidance. Advice can be obtained from HSE Head Office or from your nearest HSE Office and Knowledge Centre (www.hse.gov.uk/contact/maps/index.htm).

AC16 Dangerous Pathogens

Research organisations accommodating projects involving the use of dangerous pathogens must comply with the safeguards recommended by the Advisory Committee on Dangerous Pathogens in their guidance 'Infection at work: controlling the risk' (www.hse.gov.uk/pubns/infection.pdf), 'Biological Agents: the principles, design and operation of containment in a level 4 facility' (www.hse.gov.uk/pubns/web09.pdf) and 'Biological agents: Managing the risks in laboratories and healthcare premises' (www.hse.gov.uk/biosafety/biologagents.pdf).

AC17 Controlled Drugs

When research requires the use of one or more of the drugs controlled under the Misuse of Drugs Act, 1971 and its subsequent amendments, researchers must hold an appropriate Home Office licence in accordance with the most up to date Regulations.

AC18 Open Access Policy - Publication Repositories

To comply with the RCUK Policy on Open Access (see RGC 22 of the RCUK Terms and Conditions) the MRC requires all publications to be deposited at the earliest opportunity, and certainly within six months of publication, in Europe PubMed Central (europepmc.org/). This applies both during and after the period of funding. The condition is subject to compliance with publishers' copyright and licensing policies. Whenever possible, the article deposited should be the published version. For more information see www.mrc.ac.uk/research/research-policy-ethics/open-access-policy/

AC19 Commercial Exploitation

The research organisation should ensure that, wherever possible, the licensing of intellectual property generated from research funded by the MRC includes provision for research use by other MRC supported scientists.

Research organisations must respond to requests from the MRC to provide assurance that appropriate systems and capabilities are in place to exploit and manage intellectual property generated from MRC-funded research.

AC20 MRC Industry Collaboration Agreement

It is a condition of MRC Industry Collaboration Agreement (MICA) awards that the PI/research organisation must provide MRC Head Office with a copy of the collaboration agreement, signed by all partners, within 3 months of the date of this letter and prior to the award start date. The agreement must be consistent with the Heads of Terms submitted with the application. The grant cannot be activated, and payments, made until this document has been submitted and approved by the MRC.

AC21 Peer Review

Peer review is an integral part of the application process and ensures research of the highest calibre is funded. MRC-funded researchers are expected to contribute to this process when invited to do so, unless they have a conflict of interest (see Reviewers Handbook, www.mrc.ac.uk/documents/pdf/reviewers-handbook/), or where the research proposed is outside their expertise. We would typically expect an MRC-funded researcher to provide at least three reviews per year.
