# Supplementary material for: Exploring the potential of civic engagement to strengthen mental health systems in Indonesia (IGNITE): a study protocol
Source: Int J Ment Health Syst. 2018 Aug 25;12:49. doi: 10.1186/s13033-018-0227-x (PMC6109339; doi:10.1186/s13033-018-0227-x)
Supplement: Supplementary file 2 — Additional file 2. Ethical approval from the University of Liverpool. [file 13033_2018_227_MOESM2_ESM.pdf]

UNIVERSITY OF LIVERPOOL

Health and Life Sciences Research Ethics Committee (Psychology, Health and Society)

21 December 2017

Dear Dr Brooks

I am pleased to inform you that your application for research ethics approval has been approved. Application details and conditions of approval can be found below. Appendix A contains a list of documents approved by the Committee.

Application Details

Reference:	2715
Project Title:	Exploring the potential for civic engagement to strengthen mental health systems in Indonesia: IGNITE.
Principal Investigator/Supervisor:	Dr Helen Brooks
Co-Investigator(s):	-
Lead Student Investigator:	-
Department:	Psychological Sciences
Approval Date:	21/12/2017
Approval Expiry Date:	Five years from the approval date listed above

The application was **APPROVED** subject to the following conditions:

Conditions of approval

- All serious adverse events must be reported via the Research Integrity and Ethics Team (ethics@liverpool.ac.uk) within 24 hours of their occurrence.
- If you wish to extend the duration of the study beyond the research ethics approval expiry date listed above, a new application should be submitted.
- If you wish to make an amendment to the research, please create and submit an amendment form using the research ethics system.
- If the named Principal Investigator or Supervisor leaves the employment of the University during the course of this approval, the approval will lapse. Therefore it will be necessary to create and submit an amendment form using the research ethics system.
- It is the responsibility of the Principal Investigator/Supervisor to inform all the investigators of the terms of the approval.

Kind regards,

Health and Life Sciences Research Ethics Committee (Psychology, Health and Society)

iphsrec@liverpool.ac.uk

0151 795 5420

Appendix - Approved Documents

(Relevant only to amendments involving changes to the study documentation)

The final document set reviewed and approved by the committee is listed below:

Document Type	File Name	Date	Version
Questionnaire	Phase 2 survey_v1 24.07.17	24/07/2017	1
Interview Schedule	HSRI Interview and FG Schedules V1 2.8.17	02/08/2017	1
Fieldwork Risk Assessment	Fieldwork Risk Assessment V1 15 November 2017	15/11/2017	1
Evidence Of Peer Review	MRC Review V1 15 November 2017	15/11/2017	1
Participant Consent Form	Consent form_FGs and Interviews_SU and carer_v2 19.12.17	19/12/2017	2
Participant Consent Form	Consent form_Interview_Staff v2 19.12.17	19/12/2017	2
Advertisement	IGNITE Invitation letter v2 19.12.17	19/12/2017	2
Participant Consent Form	Consent form_Survey_v2 19.12.17	19/12/2017	2
Evidence Of Peer Review	HSRI Indonesia distress disclosure policy v3 19.12.17	19/12/2017	3
Participant Information Sheet	PIS_Survey_v2 19.12.17	19/12/2017	2
Advertisement	Recruitment poster Professionals 19.12.17 v2	19/12/2017	2
Advertisement	Recruitment poster Users and Carers 19.12.17 v2	19/12/2017	2
Participant Information Sheet	PIS_Interview_SU and carer_v2 19.12.17	19/12/2017	2
Participant Information Sheet	PIS_Interview_Staff_v2 19.12.17	19/12/2017	2
